# Changes of oxygen isotope values of soil P pools associated with changes in soil pH

**DOI:** 10.1038/s41598-020-59103-2

**Published:** 2020-02-07

**Authors:** Verena Pfahler, Andy Macdonald, Andrew Mead, Andrew C. Smith, Federica Tamburini, Martin S. A. Blackwell, Steven J. Granger

**Affiliations:** 1 Rothamsted Research, Sustainable Agriculture Sciences North Wyke, Okehampton, Devon EX20 2SB UK; 2 Rothamsted Research, Sustainable Agriculture Sciences Harpenden, Harpenden, Hertfordshire AL5 2JQ UK; 3 Rothamsted Research, Computational and Analytical Sciences, Harpenden, Hertfordshire AL5 2JQ UK; 4 NERC Isotope Geoscience Laboratory, British Geological Survey, Nottingham, NG12 5GG UK; 5 Department of Environmental System Sciences, ETH Zurich, Eschikon 33, 8315 Lindau, Switzerland

**Keywords:** Element cycles, Stable isotope analysis

## Abstract

Field data about the effect of soil pH on phosphorus (P) cycling is limited. A promising tool to study P cycling under field conditions is the ^18^O:^16^O ratio of phosphate (δ^18^O_P_). In this study we investigate whether the δ^18^O_P_ can be used to elucidate the effect of soil pH on P cycling in grasslands. Soils and plants were sampled from different fertilisation and lime treatments of the Park Grass long term experiment at Rothamsted Research, UK. The soils were sequentially extracted to isolate different soil P pools, including available P and corresponding δ^18^O_P_ values were determined. We did not observe changes in plant δ^18^O_P_ value, but soil P δ^18^O_P_ values changed, and lower δ^18^O_P_ values were associated with higher soil pH values. At sites where P was not limiting, available P δ^18^O_P_ increased by up to 3‰ when lime was applied. We show that the δ^18^O_P_ method is a useful tool to investigate the effect of pH on soil P cycling under field conditions as it highlights that different soil processes must govern P availability as pH shifts. The next challenge is now to identify these underlying processes, enabling better management of soil P at different pH.

## Introduction

Global food production relies on the use of phosphorus (P) fertilisers which are primarily derived from finite rock phosphate reserves, the supply of which is restricted by their geographic distribution and threatened by geopolitics^1^. Grasslands cover approximately 25% of the world’s ice-free land mass and are important for sustaining the increasing global demand for meat^2^. It is widely recognised that grassland productivity is strongly influenced by nitrogen (N) and P inputs^3^. The yield from agricultural grasslands is often increased through the application of inorganic P fertilisers, but excessive use of P fertilisers can lead to a soil P surplus^4^, and to detrimental environmental effects, especially on water quality. To meet the increasing agricultural demand in a sustainable way, we need to improve our understanding of P cycling in grassland soils.

Phosphorus solubility, and therefore its availability to plants, is influenced by soil biotic and abiotic processes, e.g. P uptake by microorganisms, the formation of organic P compounds, sorption onto hydrous oxides or clay silicates and precipitation of P minerals, all of which can reduce the availability of P to plants^5,6^. Plants, as well as microorganisms, can adapt to P limitation by increasing the internal use efficiency of P or by increasing P availability in the soil. This involves processes like replacing phospholipids by sulfo-galactolipids and exuding organic acids and phosphatases, respectively. Associations with mycorrhizal fungi are also a common strategy of plants to increase P availability. However, not all adaptations are understood and it is, for example, still unclear how microorganisms cope with P limitation and low soil pH at the same time^7^. P availability is often said to be highest around a neutral soil pH^8^. Below optimum pH values are a global problem due to soil acidification^9,10^ and could thus further challenge the sustainable intensification of agriculture. This pH optimum for P availability was questioned recently by Barrow^11^, citing only laboratory studies for the determination of the pH optimum. But as pointed out by Simonsson *et al*.^12^, the effect of liming on P cycling under field conditions cannot be predicted by laboratory-based studies. The Park Grass Continuous Hay experiment at Rothamsted Research (Harpenden, UK) is one of the few field experiments investigating the effect of soil pH. Park Grass contains plots that have received different lime applications for over 100 years, resulting in soils with distinct pH values^13^. Based on data from Park Grass, the effect of soil pH on plant species diversity^14^ and the microbial abundance and community structure in the soil^15^ was shown. It is however challenging to identify the most important processes behind the effects of soil pH under field conditions due to the complexity of soil-microbe-plant interactions.

We investigated if the ^18^O:^16^O ratio of phosphate (δ^18^O_P_) of soil and plant P pools can be used to obtain new insights into how P cycling varies across pH gradients under field conditions. The basis of the δ^18^O_P_ technique is that P is mainly associated with oxygen (O) in the environment. Abiotic and biotic processes involving P can lead to isotopic fractionation, i.e. the enrichment or depletion of ^18^O relative to ^16^O in case of the δ^18^O_P_. While the effect of abiotic processes in soils on the δ^18^O_P_ seems to be negligible^16–18^, δ^18^O_P_ signatures can be altered by biological processes. For example, a preference for the lighter phosphate isotopologue was observed for phosphate uptake by *Escherichia coli*, leaving the remaining phosphate enriched in ^18^O^19^. Hydrolysing enzymes, which can be excreted by microorganisms and plants, can alter the δ^18^O_P_ by cleaving the P-O bond. Fractionation factors, i.e. the extent of isotopic fractionations, of such enzymatic processes are so far known for only a few enzymes involved in the P cycle, such as acid phosphatase (−10‰)^20^ and alkaline phosphatase (−30‰)^21^. The inorganic pyrophosphatase (PPase), an ubiquitous intracellular enzyme, leads to a temperature-dependent equilibrium between O in water and in phosphate^22^. The δ^18^O_P_ values reported in the literature are often compared to this theoretical value, as it is considered to be an indication of P cycling through microorganisms^23^.

We investigate if shifts in available P δ^18^O_P_ values in the soil are associated with changes in soil management strategies, particularly changes in N applications and soil pH. We focus on available P, because it is rapidly cycled within the soil and thus greatly influenced by processes like the hydrolysis of organic P. Less mobile P pools like the mineral P pool will not be associated with variations in soil pH.

## Results

### Soil characteristics

The +L treatments always resulted in a higher pH than −L treatments (Table 1), with soil pH values as low as 3.13 (+N + P − L, 10–20 cm). Soil pH did not vary with depth while both TC and TN concentrations decreased with depth. Total C ranged between 1.3 and 10.8% dry weight (DW) and TN ranged between 0.14 and 0.85% DW. The highest TC and TN concentrations occurred in the uppermost 10 cm of the soil profile and were highest in the +N + P − L plot (Table 1).

**Table 1:** Further soil characteristics of the sampled treatments. +L and −L = with or without addition of lime, respectively. +N and −N = with or without addition of nitrogen (N) fertiliser. +P and −P = with or without addition of P fertiliser. Total C = total carbon. DW = dry weight.

Treatment	Depth (cm)	Soil pH		Total C (% DW)		Total N (% DW)	
		+L	−L	+L	−L	+L	−L
−N+P	0–10	6.40	4.37	5.08	3.81	0.49	0.34
	10–20	5.91	4.26	3.71	2.67	0.37	0.23
	20–30	6.14	4.49	1.70	1.35	0.21	0.16
+N + P	0–10	6.36	3.24	5.95	10.80	0.54	0.85
	10–20	6.29	3.13	4.22	4.31	0.39	0.37
	20–30	5.91	3.37	1.69	1.47	0.14	0.17
−N − P	0–10	6.52	4.49	4.95	4.69	0.45	0.42
	10–20	5.98	4.35	3.21	2.73	0.34	0.28
	20–30	5.95	4.74	1.60	1.33	0.19	0.18

### Phosphorus concentrations

The lime from the sample archive contained 0.5 mg P g^−1^, meaning that the +L treatment receives a minimal Pi application and that the rate in 2015 was 1.75, 1.25, and 0.875 kg P ha^−1^ for the +N + P + L, −N + P + L, and −N − P + L treatment, respectively. Variation in P concentrations in the soil P pools, except residual P, were associated with changes of depth and treatment (Table 2). Within each treatment, P concentrations of the different P pools declined with depth, with the exception of microbial P in the +N + P treatments and the −N + P − L treatment (Table 2). Except for microbial and residual P, the highest P concentrations were found in the 0–10 cm layer of the +N + P treatments (Table 2). Microbial P concentration was highest in the −N − P − L treatment and residual P concentration was highest in the −N + P + L treatment. The lowest P concentrations, except for microbial and residual P, were found in the −N − P treatments. Microbial P concentrations were lowest in the fertilised treatments and residual P concentration was lowest in the +N + P − L treatment (Table 2).

**Table 2:** Phosphorus concentrations (mg P kg^−1^ soil) of different soil P pools extracted sequentially.

Treatment	Depth (cm)	Resin P		Microbial P		NaOH-EDTA Pi		NaOH-EDTA P_org_		HCl P		Residual P		Total P	
		+L	−L	+L	−L	+L	−L	+L	−L	+L	−L	+L	−L	+L	−L
−N + P	0–10	87.7	77.2	15.9	23.2	811.4	1001.3	312.2	424.7	84.9	67.6	513.5	223.2	1825.6	1817.1
	10–20	46.9	54.8	11.3	**0.0**	710.1	712.5	356.7	265.2	61.2	28.3	287.0	217.6	1473.3	1278.3
	20–30	18.1	11.2	**0.0**	6.9	290.2	239.1	**63.9**	96.0	11.7	5.7	346.2	196.5	730.1	555.4
+N + P	0–10	57.1	126.9	**0.0**	**0.0**	903.6	1170.3	382.5	495.8	98.1	90.4	247.5	231.8	1688.8	2115.2
	10–20	22.6	37.4	5.1	18.7	662.5	1108.7	359.5	336.8	44.9	36.9	221.1	**110.8**	1315.7	1649.2
	20–30	7.6	6.3	2.2	**0.0**	284.9	301.1	156.3	118.8	15.8	11.9	298.9	342.8	765.7	780.9
−N − P	0–10	0.5	1.2	32.3	41.2	51.5	31.8	164.8	216.7	4.2	3.2	217.5	146.2	470.7	440.4
	10–20	0.4	0.6	16.0	19.0	37.4	**28.0**	159.3	163.3	3.7	**1.7**	231.9	183.9	448.7	396.5
	20–30	0.3	**0.2**	3.0	7.2	38.5	31.6	130.7	135.2	5.3	4.6	173.5	184.6	**351.3**	363.4

+L and −L = with or without addition of lime, respectively. +N and −N = with or without addition of nitrogen (N) fertiliser. +P and −P = with or without addition of P fertiliser. Bold numbers indicate the lowest concentrations for each P pool across treatments and depth, whereas underlined numbers indicate the highest concentrations.

### Calculated equilibrium values

Soil temperatures ranged from 3.3 to 4.7 °C in the 0–10 cm layer, 4.4 to 4.9 °C in the 10–20 cm layer, and 4.8 to 5.1 °C in the 20–30 cm layer in the 24 hours prior to soil sampling. The soil water δ^18^O_H2O_ ranged between −7.4 and −5.3‰ (Table 3) and decreased with depth. The calculated equilibrium values ranged between 18.3 and 20.7‰ and did not show any marked variation across the treatments.

**Table 3:** Soil water δ^18^O_H2O_, equilibrium δ^18^O_P_ range^a^, and resin and hexanol P, NaOH-EDTA inorganic P (Pi) and HCl P δ^18^O_P_. The δ^18^O_P_ values discussed within the manuscript are highlighted in bold text. nd = not determined. +L and −L = with or without addition of lime, respectively. +N and −N = with or without addition of nitrogen (N) fertiliser. +P and −P = with or without addition of P fertiliser. Contains Isotope Data, provided by the British Geological Survey, UKRI.

Treatment	Depth (cm)	Soil water δ^18^O_H2O_ (‰)		δ^18^O_P_ (‰)									
				Equilibrium range		Resin P		Hexanol P		NaOH-EDTA Pi		HCl P	
		+L	−L	+L	−L	+L	−L	+L	−L	+L	−L	+L	−L
−N + P	0–10	−5.9	−5.6	19.8–20–0	20.1–20.3	**20.0**	**23.0**	18.3	21.7	**15.2**	**17.6**	**21.7**	**22.7**
	10–20	−6.7	−6.9	19.0–19.1	18.8–18.9	**20.3**	**24.5**	19.5	23.2	nd	nd	**20.4**	**21.7**
	20–30	−6.6	−6.4	19.0–19.1	19.3–19.3	**19.7**	**21.9**	18.4	21.8	nd	nd	**19.0**	nd
+N + P	0–10	−5.5	−5.3	20.2–20.4	20.4–20.7	**18.7**	**21.9**	17.9	22.1	**20.7**	**17.4**	**18.5**	**22.2**
	10–20	−6.4	−6.2	19.2–19.3	19.5–19.6	**19.1**	**21.6**	17.9	21.5	nd	nd	**21.1**	**23.5**
	20–30	−7.4	−6.2	18.3–18.3	19.4–19.5	**18.9**	**21.2**	17.6	23.6	nd	nd	**20.9**	**23.9**
−N − P	0–10	−5.9	−5.6	19.9–20.1	20.1–20.4	nd	nd	**13.0**	**15.1**	nd	nd	nd	nd
	10–20	−7.1	−6.9	18.5–18.6	18.8–18.9	nd	nd	**14.0**	**14.1**	nd	nd	nd	nd
	20–30	−7.2	−6.9	18.4–18.5	18.7–18.8	nd	nd	nd	**15.4**	nd	nd	nd	nd

^a^Equilibrium range was calculated based on the minimum and maximum soil temperature during the 24-hour period before soil sampling for each depth: 3.3–4.7 °C (0–10 cm), 4.4–4.9 °C (10–20 cm), and 4.8–5.1 °C (20–30 cm).

### Oxygen isotope values of lime, vegetation, and soil phosphorus pools

The lime from the sample archive of Rothamsted Research had a δ^18^O_P_ of 14.7‰ (2007) and 18.0‰ (2013). The fertiliser δ^18^O_P_ was 20.6‰ (1986) and 18.6‰ (2012). The TCA P δ^18^O_P_ of the vegetation ranged between 16.3 and 22.9‰ but was not associated with the application of lime (+L vs −L: 22.9 vs 22.3‰ (−N + P), 22.3 vs 21.2‰ (+N + P), and 16.3 vs 16.4‰ (−N − P)). Similarly, the TCA P δ^18^O_P_ was not associated with the application of N fertiliser (−N + P vs + N + P treatment; mean: 22.6 vs 21.8‰), however the δ^18^O_P_ in the vegetation was lower in the −N−P treatment (mean: 16.3‰).

The soil P δ^18^O_P_ values are presented in Table 3. Resin and HCl P concentrations of the −N − P − L and −N − P + L treatments were too low for the determination of δ^18^O_P_ values. The hexanol P δ^18^O_P_ values of the −N − P treatments were less than the fertilised treatments (13.0 to 15.4‰ vs 17.6 to 23.6‰). The resin and hexanol P δ^18^O_P_, along with the concentrations of resin, microbial and hexanol P, were used to determine microbial P δ^18^O_P_ values. However, for our data this approach is not straightforward. This is because the microbial P concentration has been calculated from a function of the resin P and the hexanol P (the latter always containing the former) and corrected for sorption onto the soil. For our soils either microbial P concentrations were almost negligible (fertilised treatments) or resin P concentrations were negligible (−N − P − L and −N − P + L treatments). Thus, correcting the hexanol P δ^18^O_P_ values could lead to misleading results.

For example, the resin and hexanol P δ^18^O_P_ values in the 10–20 cm layer of the −N + P + L treatment were very similar (20.3 vs 19.5‰). However, the calculated microbial P δ^18^O_P_ value, using resin and hexanol δ^18^O_P_ values, would be 16.3‰. Given that the 0.8‰ difference between resin and hexanol δ^18^O_P_ values could largely be accounted for by experimental noise we cannot be certain that the calculated microbial P δ^18^O_P_ is correct. Given this uncertainty, and that the contribution of microbial P is negligible within the fertilised treatments, we did not attempt to calculate microbial P δ^18^O_P_ from the hexanol P δ^18^O_P_. A similar issue occurred in the −N−P treatments, whereby only low concentrations of resin P were measured as opposed to higher concentrations in the hexanol extraction. Rather than adjust the hexanol P δ^18^O_P_ values, which would cause uncertainties, we assumed that the hexanol P δ^18^O_P_ data approximated the microbial P δ^18^O_P_ in the −N − P treatments. Therefore, we will only discuss the resin P δ^18^O_P_ from the fertilised treatments and the hexanol P δ^18^O_P_ from the −N − P treatments, acknowledging the caveats above.

Variations in resin, hexanol, and HCl P δ^18^O_P_ were not associated with changes in depth within treatments, regardless of the changes in P concentrations with depth. In the fertilised treatments, resin P δ^18^O_P_ was higher in the −L treatments. This was not observed for the hexanol P δ^18^O_P_ within the −N − P treatments, but hexanol P δ^18^O_P_ was at least 5‰ lower than resin P δ^18^O_P_ in the fertilised treatments (Table 3). The HCl P δ^18^O_P_ ranged between 18.5 and 23.9‰ and showed a similar trend to the resin P δ^18^O_P_ (δ^18^O_P_ values in the +L treatments less than those in the −L treatments). For the 0–10 cm soil samples in the fertilised treatments, where the NaOH-EDTA Pi δ^18^O_P_ was measured, values ranged between 15.2 and 20.7‰. The NaOH-EDTA Pi δ^18^O_P_ were very similar in the −L fertilised treatments but differed between the +L treatments, with the NaOH-EDTA Pi δ^18^O_P_ in the −N + P treatment being around 5‰ lower than in the +N + P treatment. The NaOH-EDTA Pi δ^18^O_P_ were different from the corresponding resin and HCl P δ^18^O_P_, however while they were noticeably lower in most cases (up to −6.5‰) the δ^18^O_P_ value was higher (up to 2.2‰) in the +N + P + L treatment (Table 3).

## Discussion

The relative proportions and distribution of the soil P pools within the soil profile were typical of those reported elsewhere^24–26^. In most cases all measured P pools decreased in concentration with depth. This reflects the accumulation of P in the upper layer in soils, through the adsorption of fertilizer to soil particles and through the accumulation of organic matter, and the low mobility of P^27^. Applying N fertiliser was associated with a small decrease in P concentrations as observed elsewhere^28^. The exception was the 0–10 cm layer of the +N + P − L treatment. In this treatment, a mat of partially decomposed plant material had developed, probably resulting from the effect of the very low soil pH on microbial activity and thus carbon cycling^29^. This organic matter rich layer was removed from the soil sample, but probably influenced P concentrations as indicated by our results (Table 2).

Higher NaOH-EDTA Pi and NaOH-EDTA P_org_, concentrations were associated with the application of fertiliser and in general the NaOH-EDTA Pi pool was greater than the NaOH-EDTA P_org_ pool within the fertilised treatments. This reflects the increased input of P to these systems, which is more readily stored as NaOH-EDTA Pi than HCl P, as also indicated by the positive correlation between total P and NaOH-EDTA Pi (R^2^ = 0.96; data not shown). Changes in resin and microbial P concentrations as well as NaOH-EDTA P_org_ concentrations were only slightly associated with the application of lime, but this did result in higher NaOH-EDTA Pi concentrations in the +L treatments mainly in the upper 10 cm. This is somewhat surprising as liming increased organic P mineralisation in previous studies^30,31^, although more recent studies also found no or little effect of liming on organic P concentrations^12,32^. The +L treatments always had slightly higher HCl P concentrations compared to the −L treatments, which could be the result of the small Pi impurities in the lime. A concentration of 0.5 mg P g^−1^ lime, as found in the samples we analysed, is not unusual^33^. The lower HCl P concentrations in the −L treatments might also result from the increased solubility of HCl P, i.e. mineral P mainly, with decreasing pH. Decomposition of plant material leads to the release of Pi (represented by TCA P in our study) to the soil. Plant TCA P can have distinct δ^18^O_P_ values compared to soil P pools and leave an imprint on the δ^18^O_P_ of soil P pools^23^. Changes in the TCA P δ^18^O_P_ of the vegetation was not associated with the application of lime or N within the treatments and therefore seems to represent an impact of P fertilisation. This decrease in TCA P δ^18^O_P_ values from the P fertilised treatments (−N + P − L, −N + P + L, + N + P − L, and +N + P + L; 21.2–22.9‰) relative to the control (16.3–16.4‰) is most likely caused by an increase in leaf acid phosphatase activity in the control, which hydrolyses organic P within the leaves producing ^18^O depleted Pi, which then becomes part of the TCA P^34^.

The δ^18^O_P_ of the soil P pools varied little with depth (Table 3). This contrasts with the P concentrations and soil water δ^18^O_H2O_ values, which show a depth profile. Soil temperatures were low (around 4 °C) at the time of sampling and might have impacted biological activity and mineralization rates. As biological activity is considered a main driver of changes in δ^18^O_P_ values, this could explain the lack of a depth gradient. As the δ^18^O_P_ was not associated with changes of the sampling depth within the soil profile, the mean of the three depth values are used in the further discussion (Table 4). Hexanol P δ^18^O_P_ values for the fertilised treatments are not given in Table 4 as they are not relevant for the discussion.

**Table 4:** Resin, hexanol, and HCl P δ^18^O_P_ values (in ‰) expressed as mean over the three depths in each treatment and trichloroacetic acid soluble reactive (TCA) P δ^18^O_P_ values (in ‰) of the vegetation. Contains Isotope Data, provided by the British Geological Survey, UKRI.

Treatment		Equilibrium Range	TCA P δ^18^O_P_	Resin P δ^18^O_P_	Hexanol P δ^18^O_P_	HCl P δ^18^O_P_
−N + P	+L	18.3 to 20.7	22.9 ± 0.9	20.0 ± 0.3	*nr*	20.4 ± 1.4
	−L		22.3 ± 1.4	23.1 ± 1.3	*nr*	22.2 ± 0.8
+N + P	+L		22.3 ± 0.4	18.9 ± 0.2	*nr*	20.2 ± 1.5
	−L		21.2 ± 1.7	21.6 ± 0.4	*nr*	23.2 ± 0.9
−N − P	+L		16.3 ± 0.3	*nd*	13.5 ± 0.7	*nd*
	−L		16.4 ± 0.6	*nd*	14.8 ± 0.7	*nd*

Equilibrium range (in ‰) is the min and max equilibrium value across treatments and depth based on the min and max values of soil water δ^18^O and soil temperature of the 24 hours ahead of soil sampling. +L and −L = with or without addition of lime, respectively. +N and −N = with or without addition of nitrogen (N) fertiliser. +P and −P = with or without addition of P fertiliser. nr = not relevant. nd = not determined.

Hexanol P δ^18^O_P_ from the −N − P treatments (an approximation for microbial P; mean 13.5 and 14.8‰, +L and −L respectively) were between 3.4 and 7.2‰ lower than equilibrium, and lower than any other δ^18^O_P_ values in our study. Microbial P δ^18^O_P_ values, approximated in our study by hexanol P δ^18^O_P_ values, were also lower than the equilibrium shown in other cases^35^. Under P limitation, other intracellular processes, like the hydrolysis of organic P, could partly disturb the equilibrium inside microorganisms as observed in plants^36^. Assuming an organic P δ^18^O_P_ between 12.7 and 27‰^37^ and a soil water δ^18^O of −6.4‰, Pi released by acid or alkaline phosphatases would have a δ^18^O_P_ between 0.4 and 16.2‰, depending on the enzyme. Therefore, an increase in enzymatic organic P hydrolysis might have caused the below equilibrium hexanol P δ^18^O_P_ values.

Resin P δ^18^O_P_ values in the fertilised treatments were at or greater (approximately 3‰) than the equilibrium. This could be caused by the contribution of a P source, more enriched in ^18^O, or by a preferential uptake of the lighter Pi isotopologue by plants and/or microorganisms. However, it is questionable whether the effect of P uptake can be observed in soils, as a soil is usually a well buffered system, i.e. the resin P pool is constantly replenished from other soil P pools (mainly). It is more likely that greater than equilibrium δ^18^O_P_ values were caused by the contribution of a P source more enriched in ^18^O such as TCA P in the vegetation.

The application of N fertiliser was associated with a small decrease in resin P δ^18^O_P_ values and only slightly decreased the HCl P δ^18^O_P_, with very similar values in the case of the −N + P + L (20.4‰) and +N + P + L (20.2‰) plots (Table 4). Contrary to this, the NaOH-EDTA Pi δ^18^O_P_ only seemed to be associated with N fertilisation in −L treatments. This indicates that HCl P and NaOH-EDTA Pi δ^18^O_P_ values are influenced by processes different to those that effect the resin P δ^18^O_P_. However, the isotopic differences caused by N fertilisation are very small (<2‰; Table 4). This is in line with other studies which report also only minor effects of N fertilisation^28^. The lack of an N fertilisation effect on the δ^18^O_P_ was attributed to the fact that N limitation enhances intracellular P cycling, which should lead to δ^18^O_P_ values at or close to equilibrium. At the same time N limitation reduces the activity of hydrolysing enzymes, which may drive the δ^18^O_P_ values further from expected equilibrium.

Changes in the soil P δ^18^O_P_ values of the treatments tested (Tables 2 and 3) were mainly associated with the application of lime, with +L treatments being approximately 3‰ lower than −L. The lime had small Pi impurities (0.5 mg P g^−1^) which had δ^18^O_P_ values of 14.7 (2007) and 18.0 (2013) ‰. This however, should not be a confounding issue as applied P sources are unlikely to be directly reflected in the resin P δ^18^O_P,_ as the time between sampling and application was about one in year in our study^38^. It is more likely that the resin P δ^18^O_P_ values in the +L and −L treatments differ due to differences in P cycling between those treatments. This is also supported by the hexanol P δ^18^O_P_ values, which show a similar pattern as resin P δ^18^O_P_, indicating that microbial P cycling was also affected by soil pH. As the TCA P δ^18^O_P_ values of the vegetation were not associated with differences inliming, it is unlikely that the higher resin P δ^18^O_P_ values in the −L fertilised treatments were caused by the contribution of vegetation P directly. Abiotic processes, like the dissolution of minerals, could have influenced the resin P δ^18^O_P_ if the Pi had a distinctly different δ^18^O_P_ value compared to the resin P. Additionally, the contribution of this abiotically released Pi to the resin P pool has to be relatively high compared to the contribution of biotic processes. This seems rather unlikely in the case of Park Grass because HCl P concentrations are low and the soil pH is unfavourable for the desorption of Pi from oxides^39,40^. Furthermore, NaOH-EDTA Pi δ^18^O_P_ in the +N + P + L treatment is enriched in ^18^O compared to the +N + P − L treatment, whereas the opposite is the case for resin P δ^18^O_P_. This implies that the shift in resin P δ^18^O_P_ between the +N + P + L and + N + P − L treatment is most likely due to biotic processes like enzymatic hydrolysis and not due to abiotic processes. Lime P δ^18^O_P_ values could potentially impact HCl P δ^18^O_P,_ as these two pools are more stable than resin and microbial P. The HCl P δ^18^O_P_ values, as well as HCl P concentrations, are higher in the −L treatments compared to the +L treatments. Based on a simple mass balance, using the δ^18^O_P_ values of the lime and the HCl P of the −L treatments, the P impurities in the lime could explain the decrease in HCl P δ^18^O_P_ values from the −L to +L treatments. But the HCl P δ^18^O_P_ values are also within the reported δ^18^O_P_ range for sedimentary rocks (12–21‰^41^, the applied fertiliser (18.6 and 20.6‰ for the two fertiliser samples analysed in this study, but other fertiliser samples from the Rothamsted Sample Archive had δ^18^O_P_ values up to 22.9‰ (A. C. Smith, personal communication)), and the TCA and resin P δ^18^O_P_ values. The similarity of the values makes it difficult to disentangle the origin of the HCl P based on δ^18^O_P_. It is, however, striking that we observe the same trends in the HCl P δ^18^O_P_ values as for the resin P within the same treatments. This indicates that HCl P could be an important component of P cycling in the Park Grass soil system and it is likely there is a continuous exchange between resin and HCl P via the dissolution and formation of secondary P minerals^42,43^.

## Conclusions

Even in a complex system like the one in Park Grass, changes in soil management were associated with changes in δ^18^O_P_ values. Those changes in δ^18^O_P_ values indicate that the processes involved in P cycling vary depending on the application of lime. Neither soil depth nor nitrogen fertiliser application were as strongly associated with changes in δ^18^O_P_ values as liming. What caused those shifts remains unclear and needs further investigation. However, we have showed that the δ^18^O_P_ can be used to study the effect of soil management on P cycling under field conditions. Withholding P fertiliser also had an impact on soil P δ^18^O_P_ values, however, our data is not sufficient to draw solid conclusions about the effect of P limitation on soil P δ^18^O_P_ values.

## Methods

### Site description

The Park Grass Continuous Hay experiment was established by John Lawes and Henry Gilbert at Rothamsted (Harpenden, Herts, UK) in 1856. It was established on a site which had been in permanent pasture for at least 100 years before the experiment began^44^. It was started to test the effects of different combinations of mineral fertilisers and organic manures on the productivity of permanent grassland cut for hay. The treatments include, an unfertilised control against which different amounts and combinations of mineral fertilisers (including N, P, K, Na, Mg and farmyard manure/poultry manure) are compared^44^. In 1903 most plots were divided into two and lime (CaCO_3_) was applied to one half at 4 t ha^−1^ every four years. In 1965 most of the plots were divided further into four sub-plots, which now receive lime every third year to maintain soil pH at 7 (sub-plot ‘a’), 6 (sub-plot ‘b’), and 5 (sub-plot ‘c’), respectively. The fourth sub-plot (‘d’) does not receive lime and its pH varies depending on the treatment but is usually around pH 4 or 5. The soil is a chromic luvisol (FAO), referred to locally as a Stagnogleyic paleo-argillic brown earth of the Batcombe series^45^.

Having been designed before the pioneering research of R.A. Fisher at Rothamsted in the early 1900s, the Park Grass Experiment does not have the key statistical properties of replication and randomisation that are needed for most formal statistical analyses of data from designed experiments. Additional constraints in this study meant that samples could be collected and processed for only 6 plots with soil samples then obtained for three separate depths. Any formal statistical analysis would have to make very strong assumptions about the sources of variation, for example assessing the variation due to the main effects of the factors (fertiliser treatment, liming, depth) against the variation due to interactions amongst these factors. Any such analyses would also have low power to detect differences as statistically significant. As the aim of the study was to demonstrate the potential of the oxygen isotope ratio methodology to detect variation in P cycling responses under a range of fertiliser and pH conditions, rather than to test for differences due to these treatments, no formal statistical analysis has been performed, with observed values for the 18 treatment combinations presented to demonstrate patterns of response associated with changes in the soil system, and stimulate further study using this methodology.

### Sampling and sample preparation

Our aim was to obtain a wide range of soil pH values. We therefore included treatments with triple superphosphate and either with or without application of ammonium sulphate (plots 11/1 (referred to hereafter as “+N + P”) and 7/2 (referred to hereafter as “−N + P”)) and an unfertilised control (plot 12; referred to hereafter as “−N − P”) (Table 5). Within each of the three selected treatments, soil samples were taken from the ‘a’ and ‘d’ sub-plots. Sub-plot ‘a’, which received lime to maintain soil at pH 7, is referred to as ‘+L’ and sub-plot ‘d’, which never receives lime as ‘−L’ hereafter. Lime is applied every third year and was last applied in February 2015. Sampling took place on 1 March 2016, about one and a half months before application of N fertiliser (applied 19.4.2016) but about three months after the application of the other fertilisers including P fertiliser (applied 02.12.2015) (Table 5).

**Table 5:** Treatments of Park Grass analysed in this study including the dominant plant species (percentage >10%) and the total number of species (Sp) (data from 2000 and obtained from the electronic Rothamsted Archive). +L and −L = with or without addition of lime, respectively. + N and −N = with or without addition of nitrogen (N) fertiliser. + P and −P = with or without addition of P fertiliser.

Referred to in text	Plot	Sub-plot	Nitrogen fertiliser (kg ha^−1^ yr^−1^)	Other fertilisers (kg ha^−1^ yr^−1^)	Lime (t ha^−1^)	Dominant plant species in 2000^a^
−N + P + L	7/2	a	—	Triple superphosphate (35); potassium sulphate (225); sodium sulphate (15); magnesium sulphate (10)	2.5	*Poa trivialis* (12.8%), *Lathyrus pratensis* (15.2%), *Trifolium pratense* (10.5%); Sp = 23
−N + P − L	7/2	d	—		—	*Festuca rubra* (17.5%), *Agrostis capillaris* (48.3%); Sp = 21
+N + P + L	11/1	a	Ammonium sulphate (144)		3.5	*Poa trivialis* (46.5%), *Holcus lanatus* (15.6%), *Arrhenatherum elatius* (15.1%); Sp = 10
+N + P − L	11/1	d			—	*Holcus lanatus* (99.8%); Sp = 2
−N−P + L	12	a	—	—	1.75	*Plantago lanceolate* (11.4%), *Leontodon hispidus* (17.1%), *Briza media* (14.6%), *Lotus corniculatus* (10.1%); Sp = 30
−N − P − L	12	d	—	—	—	*Festuca rubra* (22.0%), *Agrostis capillaris* (31.9%), *Leontodon hispidus* (16.0%); Sp = 22

^a^This is the most recent data available for our selected treatments. A more recent survey of the “d” subplots showed that the dominant species in the d subplots in 2010–2012 are very similar as they were in 1991–2000 (Table 5 in Rothamsted Long-term Experiments^13^.

A mat of partially decomposed plant material had developed as a result of the low soil pH on the surface of the +N + P − L treatment. This mat was removed prior to soil sampling. From each of the selected treatments, 18 soil cores were taken by hand in a W-sampling pattern to a depth of 30 cm using a soil corer (internal diameter 2 cm). The cores were divided into three depths: 0–10 cm, 10–20 cm, and 20–30 cm. They were bulked to give one sample per depth; a total of three samples per plot. The fresh soil samples were immediately sieved <2 mm, removing large stones, visible plant material and soil fauna and ensuring complete mixing. Three subsamples were taken from each sieved and fresh soil sample: (1) approximately 10 g for extraction of soil water, stored in tightly closed vacutainers at −20 °C, (2) approximately 50 g for soil moisture content, dried at 105 °C for 24 hours; (3) approximately 10 g for the determination of soil pH, total carbon (C), nitrogen (N) and P, air-dried, milled and sieved <2 mm. The remaining sample was stored at 4 °C prior to extraction of resin and hexanol P for δ^18^O_P_. For the aboveground vegetation samples, vegetation was cut at ground level with scissors from the same locations as the soil cores and bulked per plot, giving six vegetation samples in total. From each sample, a small subsample (approximately 2 g) was stored in a tightly closed vacutainer at −20 °C for the extraction of plant water. The remaining vegetation samples were stored in plastic bags at −20 °C. Soil temperatures were obtained from the electronic Rothamsted archive (e-RA) of Rothamsted Research. The meteorological station is approximately 1 km from Park Grass. Soil temperatures are measured by Rothamsted Research hourly at a depth of 10, 20 and 30 cm in soil under grass.

### Phosphorus in plants, lime and soil

Inorganic P (Pi) from the frozen plant material was extracted with 0.3 M trichloroacetic acid (TCA) as described by Pfahler *et al*.^36^ and is afterwards referred to as TCA P. The most recently available lime samples (from 2007 and 2013) and fertiliser samples (from 1986 and 2012) from the Rothamsted Sample Archive were extracted with 1 M HCl, as described below for the soil. The following soil P pools were extracted: resin, microbial, NaOH-EDTA P_i_, NaOH-EDTA P_org_, HCl P, and residual P. The availability of those pools decreases from resin to residual P. Resin and microbial P are considered the most labile pools, which can turnover within minutes to weeks, whereas turnover rates of HCl P are estimated to be in the order of years or even millennia^35^. Within two weeks of sampling, resin, an approximation for available P, and microbial P were extracted from fresh soil with anion exchange membranes, conditioned with bicarbonate, with or without the addition of hexanol^46^. For resin P 100 g of fresh soil, and 4 resin strips (12.5 × 12.5 cm) were shaken for 16 hours at 4 °C in 5 L ultra‐pure water (ddH_2_O)^47^. For microbial P, the process was the same but with the addition of 30 ml of hexanol. The recovered resin strips were washed thoroughly with ddH_2_O to remove any attached soil particles. The resin strips were eluted by shaking overnight in 75 mL of 0.2 M HNO_3_. As the soil to solution ratios in this extraction were elevated to enable sufficient P to be collected for analysis, a separate sequential extraction was conducted for subsequent pools using the more conventional soil to solution ratio of 1:10^48^. Microbial P was extracted again from 30 g soil (dry weight equivalent) before the soil was sequentially extracted with 0.25 M NaOH – 0.05 M EDTA, targeting oxide bound inorganic and organic P, and 1 M HCl, targeting mineral P. To account for any hydrolysis of organic P or polyphosphates during the 1 M HCl step, ^18^O-labelled (three batches: δ^18^O value of 46.1‰, 14.2‰, and 22.4‰) and unlabelled 1 M HCl (two batches: δ^18^O value of −6.4‰ and −7.1‰) was used^49^. Therefore, the soil, which was recovered from the NaOH-EDTA step, was dried at 40 °C, milled to <2 mm and divided into two equal parts. One part was extracted with ^18^O-labelled 1 M HCl, the other part was extracted with unlabelled 1 M HCl. The NaOH-EDTA extract was freeze-dried and homogenised afterwards with a pestle and mortar. The freeze-dried material from selected samples was used for the determination of the δ^18^O_P_ of the inorganic P as described by Tamburini *et al*.^37^. Inorganic P (Pi) concentrations for all extracts were determined colourimetrically on an Aquachem 250 analyser using a molybdenum blue reaction^50^, while total P in the NaOH-EDTA extracts was analysed on the same equipment following oxidation with potassium persulphate. The concentration of organic P (P_org_) in the NaOH-EDTA (afterwards referred to as NaOH-EDTA P_org_) extracts was calculated as the difference between total P and inorganic P concentrations in the NaOH-EDTA extracts. The Pi in the NaOH-EDTA is afterwards referred to as NaOH-EDTA Pi. Microbial P was calculated as difference between resin and hexanol P and corrected for P adsorption^51^.

### Soil water extraction

Soil water was extracted cryogenically from sealed, pre-frozen samples at the NERC Isotope Geoscience Facility, British Geological Survey, UK, following the method described by West *et al*.^52^. Frozen samples (approximately 10 g frozen soil) were unsealed and placed in a U-shaped vacuum tube (borosilicate glass), the sample containing side of which was immersed in liquid N to ensure complete freezing and no loss of soil water. The U tube was then evacuated to a pressure of <10^−2^ mbar, removing all residual atmospherics. Once under stable vacuum, the U tube was sealed, removed from the vacuum line and the sample side of the tube placed in a furnace at 100 °C. Soil water collection was achieved by immersing the opposite side of the glass U tube in liquid nitrogen, forcing evaporating soil water to condense and collect. This set up was maintained for >1 hour to ensure complete water transfer. Soil water was collected and stored refrigerated in 1.5 ml vials with no headspace until isotope analysis.

### Further soil analyses

Sieved and air-dried soil was used to determine the soil pH in water as described by Faithfull^53^. Total carbon (TC) and total nitrogen (TN), was determined using finely ground and air-dried soil, with an elemental analyser (NA2000, Carlo Erba Instruments, Milan, Italy). Total P in the soil was determined on air-dried and milled soil via aqua regia digestion^54^. Concentrations in the total P extracts were then determined by ICP-OES.

### Oxygen isotopes in water and phosphate

Purification of the TCA, resin, microbial and HCl P extracts and precipitation of silver phosphate (Ag_3_PO_4_) followed the protocol by Tamburini *et al*.^49^. Modifications to this method were: 1 ml of concentrated H_2_SO_4_ was added during the ammonium phosphomolybdate (APM) step during the purification of the TCA, resin and microbial P extracts to facilitate the precipitation of APM. Also a few silver nitrate (AgNO_3_) crystals were added prior to precipitation of Ag_3_PO_4_ to ensure complete removal of Cl as silver chloride (AgCl). Precipitated AgCl was filtered out using 0.2 µm polycarbonate filters. Using size exclusion gel chromatography, selected freeze-dried and re-dissolved NaOH-EDTA extracts were separated in an organic and inorganic P fraction^37^. Only the inorganic P fraction of the NaOH-EDTA extraction was purified as described above. Analysis of phosphate ^18^O:^16^O (δ^18^O) was undertaken by weighing approximately 300 μg of Ag_3_PO_4_ into a silver capsule to which a small amount of fine glassy carbon powder was added^49^. The sample was converted to carbon monoxide by dropping it into a thermal conversion elemental analyser (ThermoFinnigan, Germany) at 1400 °C, the resultant CO mixed with a helium carrier gas passes through a GC column into a Delta + XL mass spectrometer (ThermoFinnigan, Germany). δ^18^O_P_ was calculated by comparison to internal Ag_3_PO_4_ laboratory standard, ALFA-1 (ALFA-1 = δ^18^O VSMOW value of 14.2‰). In the absence of an international Ag_3_PO_4_ reference material, we derived this value for ALFA-1 by comparison to the Ag_3_PO_4_ standard ‘B2207’ (Elemental Microanalysis Ltd., England), which has been measured in an inter-laboratory comparison study to have a δ^18^O value of 21.7‰ versus VSMOW. Samples were run in triplicate, with a typical precision σ ≤ 0.3‰.

Soil water δ^18^O was determined on an Isoprime Aquaprep coupled to an Isoprime 100 dual-inlet mass spectrometer (Isoprime Ltd., Cheadle, England) through a process of headspace CO_2_ equilibration with water samples. The isotope ratios are reported as δ^18^O_H2O_ values versus VSMOW, based on comparison with laboratory standards calibrated against IAEA standards VSMOW and SLAP, with analytical precision typically σ ≤ 0.05‰. The theoretical equilibrium between O in soil water and O in phosphate was calculated for each depth and treatment using a modified version of the equation given by Chang and Blake^22^: δ^18^O_P_ = −0.18 T + 26.3 + δ^18^O_H2O_, where δ^18^O_P_ is the stable oxygen isotope ratio of phosphate at equilibrium in ‰, T is the temperature in degrees Celsius and δ^18^O_H2O_ is the stable oxygen isotope ratio of the soil water in ‰. The minimum and maximum soil temperatures in the 24 hours prior to the soil sample were used for calculating the equilibrium values as it can take up to 24 hours until the equilibrium is reached^22^. The microbial P δ^18^O_P_ was determined via mass balance using the concentrations of the resin, hexanol and microbial P and the δ^18^O_P_ values of resin and hexanol P^23^.
